# Functional Exhaustion of Type I and II Interferons Production in Severe COVID-19 Patients

**DOI:** 10.3389/fmed.2020.603961

**Published:** 2021-01-27

**Authors:** Caroline Ruetsch, Vesna Brglez, Marion Crémoni, Kévin Zorzi, Céline Fernandez, Sonia Boyer-Suavet, Sylvia Benzaken, Elisa Demonchy, Karine Risso, Johan Courjon, Eric Cua, Carole Ichai, Jean Dellamonica, Thierry Passeron, Barbara Seitz-Polski

**Affiliations:** ^1^Laboratoire d'Immunologie, Centre Hospitalier Universitaire (CHU) de Nice, Université Côte d'Azur, Nice, France; ^2^Centre Méditerranéen de Médecine Moléculaire (C3M), INSERM U1065, Université Côte d'Azur, Nice, France; ^3^Unité de Recherche Clinique de la Côte d'Azur (UR2CA), Université Côte d'Azur, Nice, France; ^4^Service d'Infectiologie, Centre Hospitalier Universitaire (CHU) de Nice, Université Côte d'Azur, Nice, France; ^5^Service de réanimation, Centre Hospitalier Universitaire (CHU) de Nice, Université Côte d'Azur, Nice, France; ^6^Service de dermatologie, Centre Hospitalier Universitaire (CHU) de Nice, Université Côte d'Azur, Nice, France

**Keywords:** immunology, infectious diseases, COVID-19, interferon, personalized medicine

## Abstract

Coronavirus disease 2019 (COVID-19) caused by severe acute respiratory syndrome coronavirus 2 (SARS-CoV-2) has emerged in Wuhan in December 2019 and has since spread across the world. Even though the majority of patients remain completely asymptomatic, some develop severe systemic complications. In this prospective study we compared the immunological profile of 101 COVID-19 patients with either mild, moderate or severe form of the disease according to the WHO classification, as well as of 50 healthy subjects, in order to identify functional immune factors independently associated with severe forms of COVID-19. Plasma cytokine levels, and cytokine levels upon *in vitro* non-specific stimulation of innate and adaptive immune cells, were measured at several time points during the course of the disease. As described previously, inflammatory cytokines IL1β, IL6, IL8, and TNFα associated with cytokine storm were significantly increased in the plasma of moderate and severe COVID-19 patients (*p* < 0.0001 for all cytokines). During follow-up, plasma IL6 levels decreased between the moment of admission to the hospital and at the last observation carried forward for patients with favorable outcome (*p* = 0.02148). After *in vitro* stimulation of immune cells from COVID-19 patients, reduced levels of both type I and type II interferons (IFNs) upon *in vitro* stimulation were correlated with increased disease severity [type I IFN (IFNα): *p* > 0.0001 mild vs. moderate and severe; type II IFN (IFNγ): *p* = 0.0002 mild vs. moderate and *p* < 0.0001 mild vs. severe] suggesting a functional exhaustion of IFNs production. Stimulated IFNα levels lower than 2.1 pg/ml and IFNγ levels lower than 15 IU/mL at admission to the hospital were associated with more complications during hospitalization (*p* = 0.0098 and *p* =0.0002, respectively). A low IFNγ level was also confirmed by multivariable analysis [*p* = 0.0349 OR = 0.98 (0.962; 0.999)] as an independent factor of complications. *In vitro* treatment with type IFNα restored type IFNγ secretion in COVID-19 patients while the secretion of pro-inflammatory cytokines IL6 and IL1β remained stable or decreased, respectively. These results (a) demonstrate a functional exhaustion of both innate and adaptive immune response in severe forms of COVID-19; (b) identify IFNα and IFNγ as new potential biomarkers of severity; and (c) highlight the importance of targeting IFNs when considering COVID-19 treatment in order to re-establish a normal balance between inflammatory and Th1 effector cytokines.

## Introduction

In the beginning of December 2019 the first cases of a viral pneumonia of unknown origin were identified in Wuhan, the capital of the Hubei province in China (1, 2). The virus responsible has been identified as a new beta coronavirus now called severe acute respiratory syndrome coronavirus 2 (SARS-CoV-2) from the same family as SARS-CoV responsible for the SARS outbreak in 2003. This coronavirus which causes the new coronavirus disease (COVID-19) has since spread across the world and caused a pandemic (3, 4).

Common symptoms in patients with COVID-19 include fever, dry cough, anosmia, shortness of breath and other flu-like symptoms (3–5). Even though the majority of patients may remain completely asymptomatic or may present with only mild symptoms, 10–20% of patients progress to severe disease characterized by severe pneumonia, acute respiratory distress and multiple organ failure, requiring immediate hospitalization in intensive care units, and often leading to death (3, 6, 7). Severe clinical symptoms such as diffuse alveolar damage, thrombosis, haemophagocytosis, and immune cell depletion have been described in the subset of patients with severe COVID-19 (8). Patients suffering from diabetes, cancer or other chronic diseases are most at risk of developing a severe form (9).

To better stratify patients who might be at risk for complications, numerous studies identified biological markers of worse prognosis, such as lymphopenia, and inflammatory markers such as C-reactive protein (CRP), lactate dehydrogenase (LDH), and cytokine levels (3, 10–15).

Several authors investigated the role of different cytokines in COVID-19 patients. As a part of the immunological response to a SARS-CoV-2 infection, patients often display an aggressive and uncontrolled inflammatory response with a secretion of large amounts of pro-inflammatory cytokines such as interleukin (IL) 6, IL10 and tumor necrosis factor α (TNFα), in an event known as cytokine storm (3, 16–22). Cytokine storm is directly correlated with lung injury, multiple organ failure, and unfavorable prognosis (19).

The interplay between the innate and adaptive immune response seems to be crucial in determining the patient's evolution, characterized by an imbalance of pro- and anti-inflammatory cytokines and the subsequent dysregulation of patient's immune response (23). Interferons (IFNs) act as a key link between the innate and the adaptive immune response. Type I IFNs (IFN-α/β) are secreted by plasmacytoid dendritic cells (pDCs), while type II IFNs (IFN-γ) are predominantly produced by natural killer cells and in minor proportion by T cells and macrophages (24–26). Both type I and type II IFNs have a plethora of antiviral effects such as inducing apoptosis of infected cells and activating macrophages, natural killer (NK) cells and T lymphocytes (24–26). In COVID-19 patients, several studies have shown a dysregulation of IFNs production (14, 27, 28).

In search of new and powerful biomarkers of unfavorable outcome in COVID-19 patients, we analyzed the capability of the immune system response by the means of *in vitro* stimulation of both adaptive and innate immune cells, thus effectively mimicking a viral infection. Indeed, *in vitro* stimulation of innate and adaptive immunity cells has previously been shown to be predictive of worse outcome in other immune-related diseases (29, 30), but has to our knowledge not yet been investigated in COVID-19 patients. In this prospective, single-center study we compared the function of innate and adaptive immune cells of COVID-19 patients with either mild, moderate or severe form of the disease, aiming to underline the mechanism responsible for the dysregulation of immune response.

## Materials and Methods

### Study Design and Population

We performed a prospective cohort study at Nice University Hospital. The inclusion criteria were: (1) all adult patients admitted for COVID-19 in consultation unit (dermatology or infectious diseases unit), in infectious diseases units or in intensive care unit, in Nice University Hospital, from March to April 2020; (2) not having received immunosuppressive therapy in the 6 months prior to inclusion; (3) ability to sign an informed consent. Exclusion criteria were: (1) all patients under 18; (2) patients under custody, in prison or with a mental illness; (3) pregnant or breastfeeding; (4) with a known immunodeficiency or having received previous immunosuppressive therapy. Fifty healthy donors not infected with SARS-CoV-2 were also recruited (confirmed by negative serological test).

According to the severity of infection with SARS-CoV-2, the patients were divided in three groups: (a) patients with a severe form of COVID-19 were those hospitalized or transferred in the intensive care unit with respiratory distress or respiratory failure requiring mechanical ventilation or multiple organ failure; (b) patients with a moderate from of COVID-19 were patients hospitalized in the infectious diseases units, defined by clinical symptoms associated with dyspnea and radiological findings of pneumonia on thoracic CT scan; (c) COVID-19 patients with mild symptoms of COVID such as chilblains in fingers and toes or flu-like symptoms not requiring hospital supervision. All patients presented a COVID-19 symptomatology according to WHO classification with a CT scan characteristic of COVID-19 (31) or chilblains (32) or two consecutive positive RT-PCR tests for SARS-CoV-2 on upper and lower respiratory tract specimens (nasopharyngeal swab or invasive respiratory sample) or positive serological test (Euroimmun® ELISA).

Epidemiological, biological and clinical data at day 0 (D0) are reported in Table 1 and Supplementary Table 1. Treatment(s) received after D0 are summarized in Supplementary Table 2. Complications were defined as all adverse events such as admission in intensive care unit after worsening of the symptoms, mechanical ventilation, deep vein thrombosis, secondary bacterial infection, kidney failure, hepatitis, heart failure and death.

**Table 1:** Demographics and baseline characteristics of healthy donors and of patients with COVID-19.

	**All cases**	**Healthy donors**	**Mild cases**	**Moderate cases**	**Severe cases**	***P*-value**
	***n* = 151**	***n* = 50**	***n* = 41**	***n* = 30**	***n* = 30**	
Age (years)	51 (36; 62)	43 (36; 53)	31 (21; 49)	65 (53; 76)	66 (54; 72)	<0.0001
Sex ratio (M/F)	66/85	11/39	17/24	17/13	21/9	0.0002
Co-morbidities (Y/N)	72/23^*a*^	NA	23/17^*b*^	27/3	22/3^*a*^	0.0018
BMI	24.1 ± 4.6	NA	22.1 ± 3.5	25.2 ± 4.3	26.5 ± 4.9	0.0003
Days after first signs of COVID-19	11 (7; 17)	NA	15 (10; 24)	9 (5; 13)	9 (5; 12)	0.0001
Lymphocytes (count/mm^3^)	1.5 (1.0; 1.9)	NA	1.8 (1.6; 1.3)	1.2 (0.8; 1.7)	1.0 (0.8; 1.2)	<0.0001
Monocytes (count/mm^3^)	0.5 (0.4; 0.7)	NA	0.5 (0.4; 0.6)	0.5 (0.4; 0.8)	0.5 (0.3; 0.7)	0.2957

*Data are presented as the median (IQR), average ± SD, or n. Global p-values comparing the differences between the groups are reported and are from one-way ANOVA, Chi-square and Kruskal-Wallis test, as appropriate. BMI, body mass index; NA, not available*.

a *Data missing for six patients and 51 healthy donors*.

b *Data missing for one patient*.

c *Data missing for five patients*.

An informed consent was obtained for all patients. The study protocol conformed to the ethical guidelines of the 1975 Declaration of Helsinki and was approved by the appropriate institutional review committee (NCT04355351).

### Blood Collection and Cytokine Assay

Blood samples were collected at D0 and several follow-up time points up to 2 months after admission to the hospital. One milliliter of whole blood was stimulated with immune ligands (anti-CD3 as T-cells stimulant, and R848 as Toll-like receptors 7/8 (TLR 7/8) agonist) on single lyophilized spheres (LyoSphere^TM^, Qiagen) within 8 h from blood collection. Stimulated blood samples were incubated for 16–24 h at 37°C and then centrifuged at 2,000 to 3,000 × g for 15 min to harvest the stimulated serum. Non-stimulated serum and plasma and stimulated serum were stored at −20°C until the analysis and freeze-thaw cycles were minimized to preserve the quality of the samples. Serum and plasma levels of cytokines with or without non-specific stimulation were measured using either QuantiFERON-Monitor test for the detection of IFN-γ, or custom-designed cartridges Ella (ProteinSimple) for the detection of IL-1β, IL-6, IL-8, IL-10, IL-17A, TNF-α, and IFN-α, following the manufacturers' instructions.

### Studies *in vitro*

For 18 COVID-19 patients, one milliliter of whole blood taken at D0 was pretreated with different molecules for 6 h at 37°C, followed by stimulation with immune ligands on single lyophilized spheres (LyoSphere^TM^, Qiagen), as described under Blood collection and cytokine assay. The molecules used were those commonly administered to COVID-19 patients (33–43): hydroxychloroquine (100 μM, Inresa), anti-IL6 Tocilizumab (100 μg/mL, RoActemra, Roche), methylprednisolone (20 μg/mL, Mylan), anti-TNFα Adalimumab (10 μg/mL, Humira, AbbVie), recombinant human IL-2 (6 ng/mL, Sigma), recombinant human IFN-alpha (100 ng/mL, Sigma) and Nivolumab (1 μg/mL, Opdivo, Bristol Myers Squibb).

### Statistics

For descriptive statistics, data are presented as mean and standard deviation for continuous values with Gaussian distribution, as median and range for continuous values with non-Gaussian distribution, and as counts and percentages for categorical variables. The D'Agostino & Pearson normality test was used to determine if a variable had a Gaussian distribution or not. Groups of continuous values were compared by the Mann-Whitney test, one-way ANOVA (>2 groups), or Kruskal-Wallis test (>2 groups). Multiple comparison tests were performed with Kruskal-Wallis test using Dunn's *post hoc* test. Categorical variables were compared using Chi-square test. AUC (Area Under the Curve) ROC (Receiver Operating Characteristic) curve was used to define an IFN-γ threshold that best discriminates patients with or without complications. Log-rank test was used to compare survival data. A Wilcoxon matched pairs signed rank test was used to compare two measurements of a continuous variable performed on the same subjects (paired data). Logistic regression were performed to determine ODDS ratios and 95% confidence intervals (CI). In the mulitivariable model, we adjusted for age, sex and BMI.

Statistical analyses were performed using GraphPad Prism 7.0 (GraphPad Software, Inc., San Diego, CA) or SAS 9.4. All comparisons were two-tailed, and the differences were considered significant when *P*-value < 0.05.

## Results

### Study Cohort

A total of 101 patients with a symptomatology of COVID-19 infection (Table 1) were included and divided in three groups based on the severity of their symptoms into mild (*n* = 41), moderate (*n* = 30) and severe cases (*n* = 30), as described in Methods. Fifty healthy donors were also recruited. As described previously (1, 3, 4), there was a significant difference in age, gender, BMI and number of comorbidities among the three groups of patients (*p* < 0.0001, *p* = 0.0002, *p* = 0.0003, and *p* = 0.0018 respectively). Most common symptoms of COVID-19 infection included cough, dyspnea and fever in 52, 52, and 42% of patients, respectively (Supplementary Table 1).

### Cytokine Levels in Non-stimulated Plasma

As expected, higher plasma levels of pro-inflammatory cytokines IL1β, IL6, IL8, and TNFα at admission and before specific treatment were positively correlated with the severity of COVID-19 symptoms (*p* < 0.0001 for all cytokines) (Table 2), confirming the results from previous studies (3, 16–18).

**Table 2:** Non-stimulated plasma cytokine levels of healthy donors and of patients with COVID-19, at baseline.

	**All cases**	**Healthy donors**	**Mild cases**	**Moderate cases**	**Severe cases**	***P*-value**
	***n* = 151**	***n* = 50**	***n* = 41**	***n* = 30**	***n* = 30**	
Plasma IL-1β (pg/mL)	0.1 (0.0; 0.2)	0.0 (0.0; 0.1)	0.1 (0.0; 0.1)	0.2 (0.1; 0.2)	0.3 (0.2; 1.0)	<0.0001
Plasma IL-6 (pg/mL)	1.5 (0.8; 12.6)	1.1 (0.7; 1.8)	0.8 (0.7; 1.3)	25.3 (4.3; 43.8)	53.7 (15.9; 74.3)	<0.0001
Plasma IL-8 (pg/mL)	3.1 (2.3; 8.7)	2.7 (2.2; 3.6)	2.4 (1.8; 2.9)	6.4 (4.0; 19.0)	13.6 (8.7; 17.9)	<0.0001
Plasma TNFα (pg/mL)	7.0 (5.7; 10.6)	6.2 (5.3; 7.3)	5.9 (5.2; 6.8)	11.9 (8.1; 15.5)	13.8 (10.6; 19.8)	<0.0001
Plasma IL17A (pg/mL)	0.0 (0.0; 0.0)	0.0 (0.0; 0.0)	0.0 (0.0; 0.0)	0.0 (0.0; 0.0)	0.0 (0.0; 0.0)	>0.9999
Plasma IFNα (pg/mL)	0.0 (0.0; 0.0)	0.0 (0.0; 0.0)	0.0 (0.0; 0.0)	0.0 (0.0; 0.0)	0.0 (0.0; 0.0)	>0.9999
Plasma IFNγ (IU/mL)	0.0 (0.0; 0.0)	0.0 (0.0; 0.0)	0.0 (0.0; 0.0)	0.0 (0.0; 0.0)	0.0 (0.0; 0.0)	>0.9999

*Data are presented as the median (IQR). Global p-values comparing the differences between the groups are from Kruskal-Wallis test. IL, interleukin; IFN, interferon; NA, not available; ND, not detected; TNF, tumor necrosis factor*.

### Cytokine Levels in Serum After *in vitro* Non-specific Stimulation of Innate and Adaptive Immunity Cells

**Figure 1:**
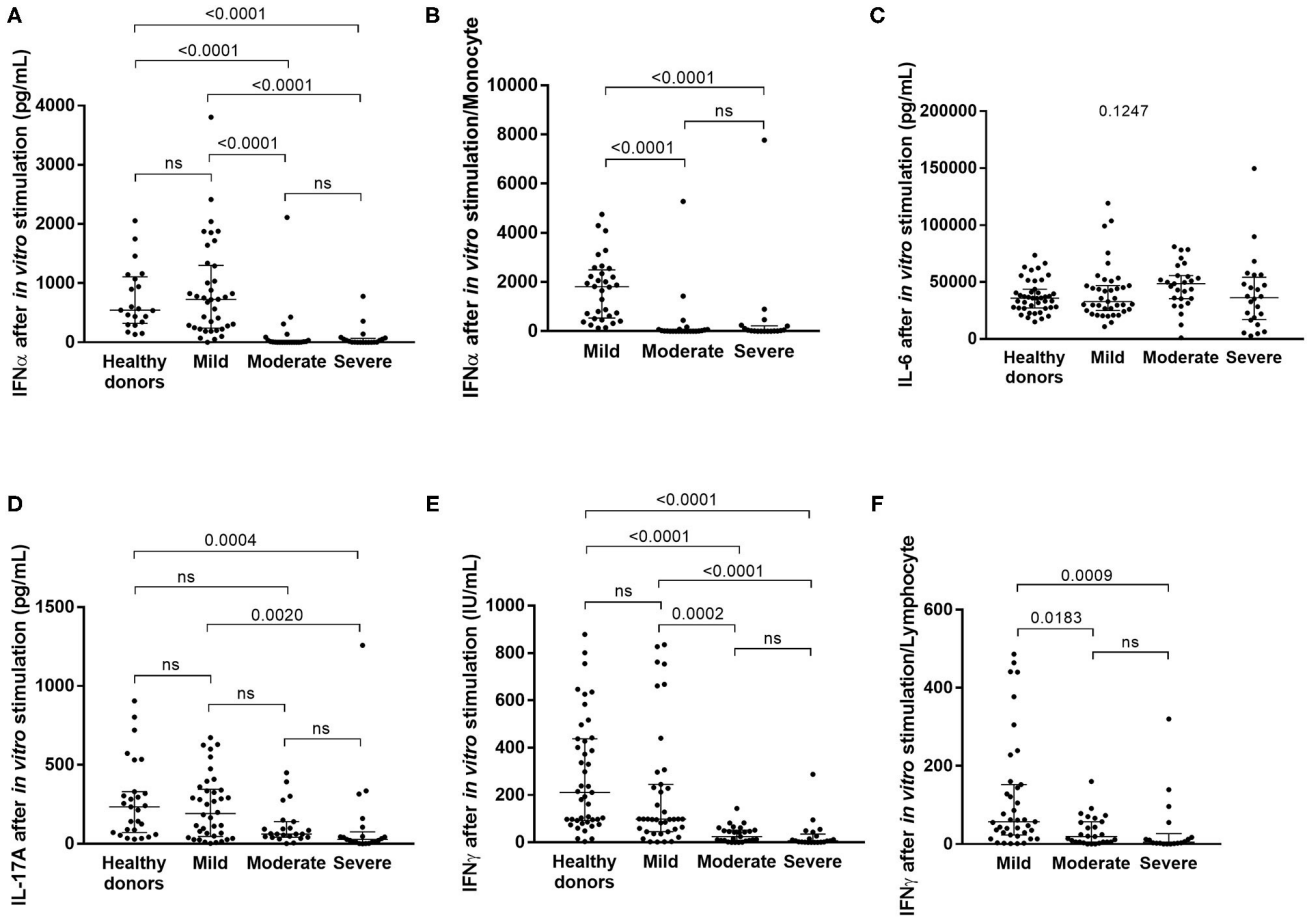
Serum cytokine levels after *in vitro* stimulation of innate and adaptive immune cells in healthy subjects and in COVID-19 patients with mild, moderate or severe symptoms. The levels of IFNα (**A**: in mild COVID-19 3 points are missing, in moderate COVID-19 3 points are missing and in severe COVID-19 patients 6 points are missing), IL6 (**C**: 3, 3, and 6 points, respectively), IL17 (**D**; 3, 7, and 9 points, respectively) and IFNγ (**E**: 2, 3 and 9 points, respectively) were measured, and the levels of IFNα and IFNγ were corrected for monocyte **(B)** and lymphocyte **(F)** count, respectively. Differences between groups were compared with Kruskal-Wallis test using Dunn's *post hoc* test.

While the current state of inflammatory response to SARS-CoV-2 infection, as evidenced by the plasma cytokine levels, reflects the ongoing interplay between innate and adaptive immunity, it tells us little about immune function. To this end, we stimulated innate cells and T lymphocytes of COVID-19 patients at admission and before specific treatment with Toll-like receptor 7/8 (TLR 7/8) agonist and anti-CD3, respectively, and we measured the cytokines secreted. TLR7 is predominantly expressed in plasmacytoid dendritic cells (pDC) (44) and TLR8 is more strongly expressed in myeloid dendritic cells, monocytes and to a lesser extent in pDC (45). Th17 cytokine IL17A, as well as type I and type II IFN were not detectable in non-stimulated plasma of COVID-19 patients (Table 2). However, after *in vitro* stimulation of immune cells significant differences in cytokine levels emerged between patients with various severity of COVID-19 (Table 3, Figure 1), reflecting the fitness of their immune system. On the innate immunity side, DCs and NK cells of moderate and severe patients were functionally exhausted as illustrated by lower IFNα (and IFNγ from NK cells) levels upon *in vitro* stimulation (*p* < 0.0001) (Figure 1A), as previously suggested (46), and the differences remained significant after correction for monocyte count (*p* < 0.0001 mild vs. moderate and mild vs. severe) (Figure 1B). Levels of IL6, which is secreted by cells of both innate and adaptive immunity, remained unchanged between groups upon *in vitro* stimulation (*p* = 0.1247) (Figure 1C). On the adaptive immunity side, functional exhaustion was observed for Th17 lymphocytes producing IL17A in severe COVID-19 patients in comparison to mild forms and healthy subjects (*p* = 0.0004 and *p* = 0.002 respectively) (Figure 1D). Strikingly, lower secretion of IFNγ correlated with increased severity of COVID-19 (*p* < 0.0001) (Figure 1E). This lower production of IFNγ remained significant even when corrected for lymphocyte count (*p* = 0.0183 mild vs. moderate and *p* = 0.0009 mild vs. severe) (Figure 1F).

**Table 3:** Serum cytokine levels after non-specific stimulation of T lymphocytes and DCs, in healthy donors and in patients with COVID-19, at baseline.

	**All cases**	**Healthy donors**	**Mild cases**	**Moderate cases**	**Severe cases**	***P*-value**
	***n* = 151**	***n* = 50**	***n* = 41**	***n* = 30**	***n* = 30**	
Stimulated IL1β (pg/mL)	2,850 (1,846; 4,701)	3,819 (2,820; 5,407)	3,226 (1,930; 4,723)	2,241 (1,254; 3,824)	1,918 (1,252; 3,208)	0.0001
Stimulated IL6 (pg/mL)	36,792 (26,906; 51,355)	35,922 (27,333; 43,741)	32,890 (25,031; 46,975)	48,567 (35,469; 55,791)	36,263 (17,096; 54,257)	0.1247
Stimulated IL8 (pg/mL)	34,869 (22,395; 65,475)	30,284 (22,371; 39,873)	28,386 (15,100; 55,727)	62,032 (33,230; 135,877)	69,042 (33,065; 133,094)	<0.0001
Stimulated TNFα (pg/mL)	6,537 (4,435; 10,538)	9,844 (6,222; 13,167)	7,461 (6,089; 13,202)	4,571 (2,372; 6,420)	3,003 (1,162; 8,262)	<0.0001
Stimulated IL17A (pg/mL)	97 (37; 299)	234 (72; 331)	192 (46; 346)	62 (42; 140)	28 (10; 76)	0.0002
Stimulated IFNα (pg/mL)	262 (13; 778)	544 (321; 1,109)	724 (241; 1,303)	6 (0; 37)	12 (1; 70)	<0.0001
Stimulated IFNγ (IU/mL)	82 (15; 230)	211 (93; 438)	98 (46; 245)	24 (8; 52)	7 (1; 36)	<0.0001

*Data are presented as the median (IQR). Global p-values comparing the differences between the groups are from Kruskal-Wallis test. DC, dendritic cell; IL, interleukin; IFN, interferon; NA, not available; TNF, tumor necrosis factor*.

### Correlation Between IFNs Production After *in vitro* Stimulation and COVID-19-Related Complications

**Figure 2:**
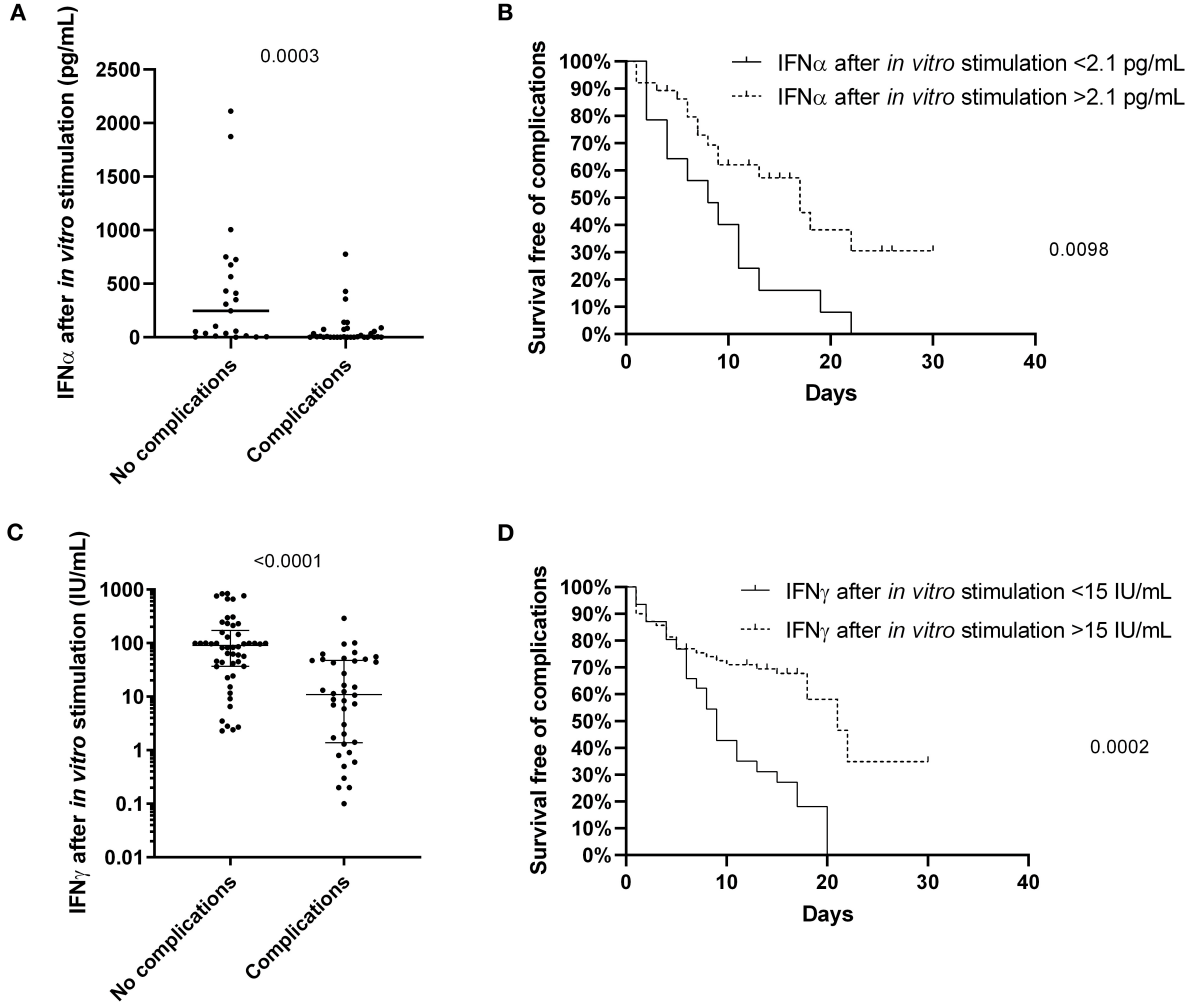
The predictive value of IFNα and IFNγ after non-specific stimulation of innate and adaptive immune cells for the patients with COVID-19. The level of IFNα (**A**; *n* = 55) and IFNγ (**C**; *n* = 89) differs between the patients with and without complications, and predicts the survival free of complications for patients with COVID-19 **(B,D)**. Differences between groups were compared with Mann-Whitney test, and log-rank test was used to compare survival data.

The level of IFNα and IFNγ production upon *in vitro* stimulation of innate and adaptive immunity cells at admission and before specific treatment was predictive of the risk of complications (*p* = 0.003 and *p* < 0.0001, respectively) (Figures 2A,C). Indeed, patients with a level of IFNα and IFNγ lower than 2.1 pg/mL and 15 IU/mL, respectively, as defined by a ROC curve (data not shown), were more likely to develop complications during hospitalization (*p* = 0.0098 and *p* = 0.0002, respectively) (Figures 2B,D). As confirmed also by multivariable analysis (Table 4), stimulated IFNγ levels are an independent predictor of complications in patients with COVID-19 [*p* = 0.0349 OR = 0.98 (0.962; 0.999)].

**Table 4:** Multivariable analysis for the evaluation of the relationship between considered variables at baseline and complications.

	**Odds ratio (95% CI)**	***P*-value**
Age (years)	1.041 (0.977–1.110)	0.2157
Gender (M/F)	4.119 (0.466–36.402)	0.2029
BMI	1.218 (0.966–1.536)	0.0954
Plasma IL6 (pg/mL)	1.072 (1.015–1.133)	0.0128
Stimulated IFNγ (pg/mL)	0.980 (0.962–0.999)	0.0349

*BMI, body mass index; IL, interleukin; IFN, interferon*.

### The Evolution of Cytokine Levels Depending on Clinical Outcome

**Figure 3:**
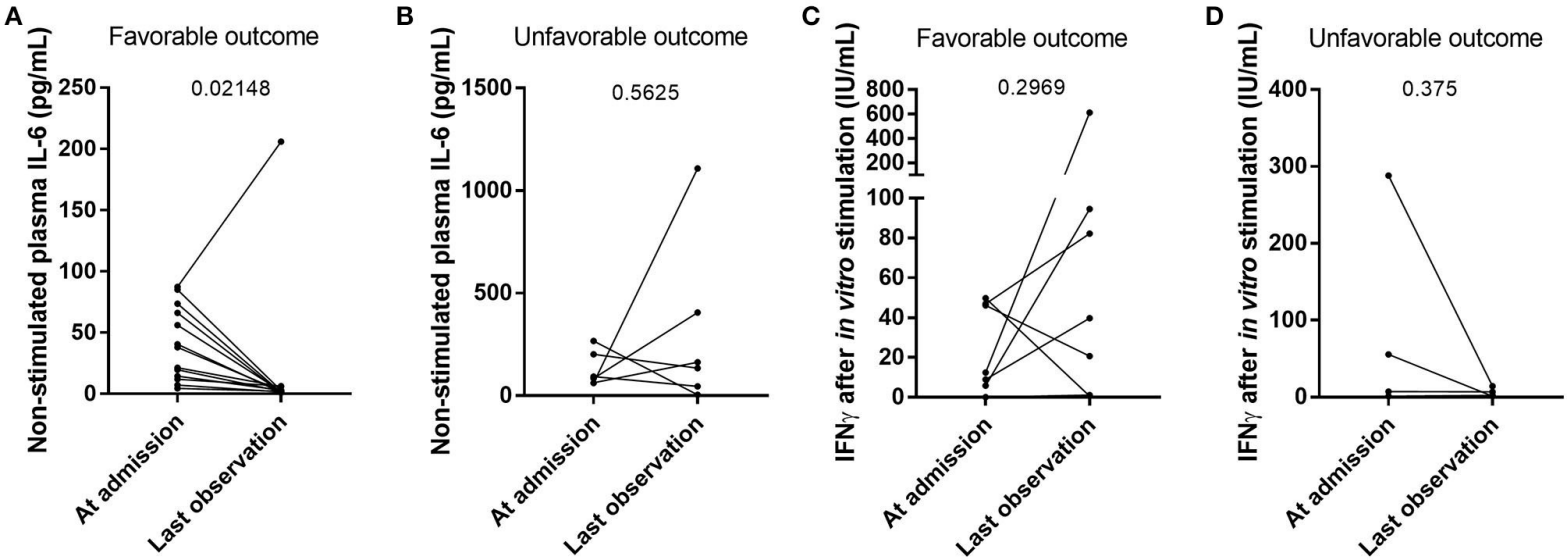
The evolution of cytokine levels in individual patients following their clinical outcome. The levels of IL6 in non-stimulated plasma (**A,B**; *n* = 13 and *n* = 6, respectively) and the levels of IFNγ after *in vitro* non-specific stimulation of innate and adaptive immune cells (**C,D**; *n* = 6 and *n* = 3, respectively) were compared between the patients who recovered from their SARS-CoV-2 infection **(A,C)** and the deceased patients **(B,D)**. Differences between groups were compared with a Wilcoxon matched pairs signed rank test.

We further assessed the evolution of cytokine production and IFN response during hospitalization in moderate and severe cases. During follow-up, non-stimulated plasma IL6 levels decreased between the moment of admission to the hospital and at the last observation carried forward for patients with favorable outcome (*p* = 0.02148) (Figure 3A and Supplementary Figure 1A), while they remained high in deceased patients (*p* = 0.5625) (Figure 3B and Supplementary Figure 1B). The level of IFNγ after *in vitro* stimulation, however, did not significantly differ between the time of admission to the hospital and the last observed time point (Figures 3C,D), which was likely due to a small number of patients per group. Two individual cases were chosen to better demonstrate the evolution of cytokine production during the course of the disease. The first case resulted in recovery with an increased stimulated IFNγ levels at the last point (Supplementary Figure 1A), while the second case resulted in death with stable low stimulated IFNγ levels throughout hospitalization (Supplementary Figure 1B).

### Effect of *in vitro* Treatment With Therapeutic Molecules on the Restoration of Cytokine Balance

**Figure 4:**
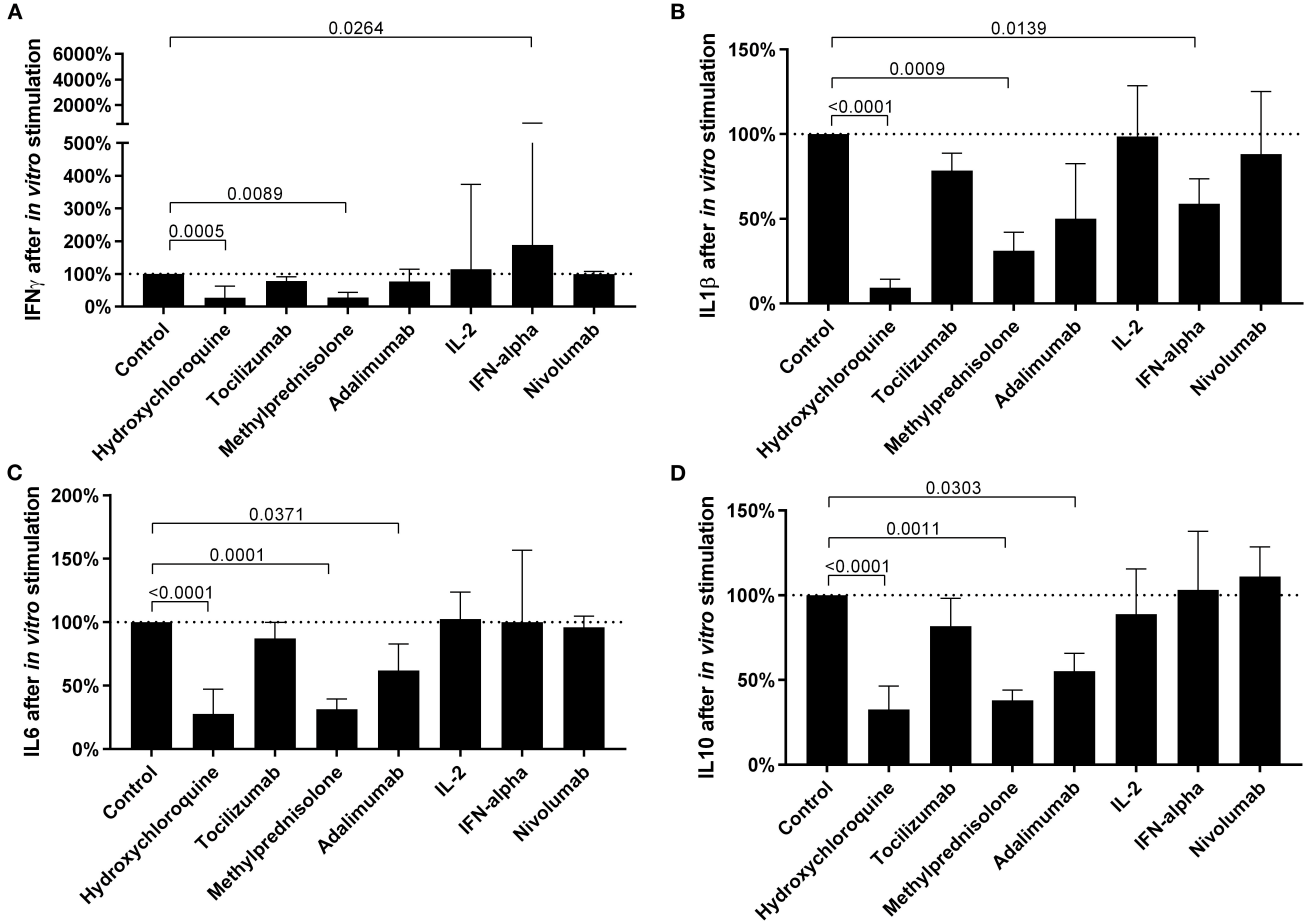
The efficacy of *in vitro* treatment with different drugs commonly used in COVID-19 to modulate cytokine expression. The levels of IFNγ **(A)**, IL1β **(B)**, IL6 **(C)**, and IL10 **(D)** after *in vitro* pretreatment with drugs, followed by non-specific stimulation of innate and adaptive immune cells, in 18 COVID-19 patients. Differences between groups were compared with Kruskal-Wallis test using Dunn's *post hoc* test.

Several drugs commonly used to treat COVID-19 patients were tested for their potential to restore cytokine balance *in vitro*, notably to increase IFNγ production and decrease the production of inflammatory cytokines, while keeping the secretion of regulatory cytokines constant. Chloroquine and methylprednisolone proved efficient in reducing secretion of all cytokines (Figure 4) while Adalimumab reduced only IL6 and IL10 secretion. Interestingly, IFNα had a more balanced effect with a strong stimulation of IFNγ and a decrease of inflammatory cytokine IL1β, while the secretion of T regulatory cytokine IL10 and pro-inflammatory cytokine (IL6) remained unchanged. The individual results are detailed in Supplementary Table 3.

## Discussion

We report here a cohort of 101 patients with a symptomatology of COVID-19 infection. We aimed to reveal their specific immunological profiles and to correlate them to the evolution and the extent of symptoms in individual patients. Our results confirmed a functional exhaustion of type I (NK cells and DCs) and type II IFN (T cells) production in moderate and severe patients traducing an evasion of both innate and adaptive immune response and in accordance with recent studies (14, 16, 18, 21, 47–49).

It is well-known that the innate immune response is triggered by virally infected cells which can be recognized by host pattern-recognition receptors (PRRs) expressed by DCs that produce a variety of cytokines (50) such as type I IFN, which in turn recruit lymphocytes and monocytes to inflamed sites (51–54). Type I IFN primarily activates epithelial cells and reduces the mononuclear macrophage-mediated proinflammatory activity (55). Type II IFNs have different functions, eliciting T helper 1 (Th1)-driven immune responses, and also enabling induced regulatory T (Treg) cells to control and regulate immune responses (56). Consequently, SARS-CoV-2 has evolved several mechanisms to inhibit type I IFN induction and signaling (57). During SARS-CoV-2 infection, both innate and adaptive immune response are required for successful virus clearance and must be adequately controlled to minimize immunopathological damage (57). By assessing the response of immune cells of infected patients after stimulation, we demonstrate here a marked decrease in type I and type II IFN response from mild to severe patients. The molecular mechanism(s) of this IFN evasion remain to be confirmed, however, several studies have suggested different pathways that could contribute to the decreased amount of IFNs in severe COVID-19 patients, from concealed viral production invisible to PPARs to direct synthesis of structural and nonstructural viral proteins that antagonize IFN signaling (47–49). Indeed, SARS-CoV-2 induced an aberrant type-I IFN response in cultured cells, characterized by a delayed antiviral response which may provide a window for virus replication and an improper recruitment of inflammatory monocyte macrophage populations (21).

The originality of our work lies in the stimulation of TLR7 and TLR8 which reproduce *in vitro* a viral infection by the activation of innate immune system and produce type I IFN (58). On the other hand, the stimulation of T lymphocytes by an anti-CD3 allowed us to quantify the production of type II IFN and to evaluate the adaptive immune response. The innate immune recognition of virus infection triggers antiviral immune responses by residual genomic RNA recognized by PRR expressed mainly by DCs (59). In moderate and severe COVID-19 patients we observed that innate cells produce less type I IFN, and consequently NK cells produce less type II IFN. In accordance with previous studies (21, 60), our results suggest that an uncontrolled infection maintains monocyte and macrophagic activation, and that the regulatory T lymphocytes remain inactivated due to a weak production of type II IFN, thus reinforcing the cytokine storm and leading to severe complications in patients with COVID-19.

This longitudinal study allowed us to conclude that a functional analysis of IFN production at the beginning of the hospitalization is a powerful tool to predict the clinical evolution of patients infected with SARS-CoV-2. While previous studies demonstrated opposing results with either impaired (14, 28) or increased (61) type I IFN response in severe COVID-19 patients, our results tip the balance toward impaired IFN signaling. According to our study on stimulated IFN production as well as in other studies (14, 21), type I IFN plays a major role in the activation of type II IFN and represents a strategic target for early treatment of COVID-19 patients, in order to destroy the immune evasion caused by SARS-CoV-2 and to treat the specific immune dysfunction.

However, there is increasing evidence that patients with severe COVID-19 may have a robust type I IFN response, which contrasts the delayed, possibly suppressed, IFN response seen early in infection (27, 62). While limited by a small sample size of eight and seven patients, respectively, Zhou et al. and Wilk et al. demonstrated that many IFN-stimulated genes are overexpressed in COVID-19 patients (62, 63).

Since more studies are needed to further illuminate the role of IFNs in COVID-19, both from a clinical and a molecular perspective, IFN treatment remains controversial as well. Nevertheless, an *in vitro* study on cultured cells has shown a potential benefit of IFNβ treatment (48), as well as a recent clinical study NCT04276688 with favorable outcome for IFNβ (33), while others are still in progress. Two recent retrospective studies found that IFNα treatment may be beneficial for COVID-19 patients (37, 38), however it seems that adequate timing in IFN administrating is crucial for its efficacy since early administration decreased mortality, while late administration had an opposite effect (38).

Our study presents several limitations. First, this is an observational study showing an association between IFN I and II levels and COVID-19 severity and outcome. Randomized clinical trials using functional interferon assays at admission to predict outcome are needed to clearly evaluate the efficacy and utility of IFN measurement in clinical practice. Second, while our *in vitro* tests and several recently published studies (33, 37, 38, 48) show the potential of IFNα treatment in order to restore the cytokine balance, the results nevertheless need to be confirmed in large controlled clinical trials. Third, this is a study on a relatively small number of patients that needs to be confirmed in larger cohorts. Notably, there were only six deaths in our cohort of COVID-19 patients, severely limiting the power of statistical analyses. Fourth, while male gender, older age and obesity have been shown to be strongly associated with increased mortality in COVID-19 patients (10, 11), low number of deaths prevented us from identifying these factors in our cohort. Instead, we tested the predictive power of age, gender and BMI on COVID-19-related complications, but apart from plasma IL6 levels and stimulated IFNγ levels the other variables remained non-significant at multivariable analysis.

The variability of symptomatology lies at the heart of our cells, among the immune responses involved in fighting infection by the COVID-19. IFNγ represents a predictive biomarker of the evolution of SARS-CoV-2 which can be safely and routinely measured in laboratory by QuantiFERON Monitor. It could allow clinicians to provide adjusted treatment and medical care in this epidemic context. Based on our results, our functional test could be an important tool to predict severe COVID-19 and guide personalized therapy targeting the immune restoration of NK and T-cells (inhibiting check-point inhibitor) and IFN production.

## Data Availability Statement

The raw data supporting the conclusions of this article will be made available by the authors, without undue reservation.

## Ethics Statement

The studies involving human participants were reviewed and approved by CPP SUD-OUEST et OUTRE-MER I. The patients/participants provided their written informed consent to participate in this study.

## Author Contributions

CR, VB, MC, SB, and BS-P designed the study. VB and MC carried out experiments. KZ, CF, CR, and MC collected clinical data. BS-P, CR, VB, and MC analyzed and interpreted the data. BS-P, CR, ED, KR, JC, EC, CI, JD, and TP provided medical oversight. BS-P, CR, and VB drafted and revised the manuscript. All authors contributed to the article and approved the submitted version.

## Conflict of Interest

The authors declare that the research was conducted in the absence of any commercial or financial relationships that could be construed as a potential conflict of interest.
