# Supplementary material for: Functional Exhaustion of Type I and II Interferons Production in Severe COVID-19 Patients
Source: Front Med (Lausanne). 2021 Jan 27;7:603961. doi: 10.3389/fmed.2020.603961 (PMC7873370; doi:10.3389/fmed.2020.603961)
Supplement: Supplementary file 1 [file Data_Sheet_1.docx]

# **Supplemental material**

**Supplementary table 1**: Symptoms of patients with COVID-19, at admission to the hospital

|  | **All cases**  **n=101** | **Mild cases**  **n=41** | **Moderate cases**  **n=30** | **Severe cases n=30** |
| --- | --- | --- | --- | --- |
| Cough | 53 (52%) | 16 (33%) | 22 (73%) | 15 (50%) |
| Fever | 42 (42%) | 8 (16%) | 19 (63%) | 15 (50%) |
| Dyspnea | 52 (52%) | 9 (18%) | 21 (70%) | 22 (73%) |
| Headache | 23 (23%) | 15 (31%) | 5 (17%) | 3 (10%) |
| Anosmia | 13 (13%) | 4 (8%) | 5 (17%) | 4 (13%) |
| Diarrhea | 26 (26%) | 10 (20%) | 9 (30%) | 7 (23%) |
| Chilblains | 31 (31%) | 31 (63%) | 0 (0%) | 0 (0%) |

Data are presented as n (%). COVID-19, coronavirus disease 2019.

**Supplementary table 2**: Treatment of patients with COVID-19, after admission to the hospital

|  | **All cases**  **n=101** | **Mild cases**  **n=41** | **Moderate cases**  **n=30** | **Severe cases**  **n=30** |
| --- | --- | --- | --- | --- |
| Anti-viral | 11 (11%) | 0 (0%) | 3 (10%) | 8 (27%) |
| Hydroxychloroquine | 9 (9%) | 1 (2%) | 3 (10%) | 5 (17%) |
| Corticosteroids | 15 (15%) | 0 (0%) | 4 (13%) | 11 (37%) |
| Antibiotics | 29 (29%) | 3 (6%) | 9 (30%) | 17 (57%) |

Data are presented as n (%). COVID-19, coronavirus disease 2019.

**Supplementary table 3**: Clinical and biological data of patients tested in Figure 4

| Patients | COVID-19 severity group | Age | Sex | Co-Morbidities (Y/N) | Plasma IL-1β (pg/mL) | Plasma IL-6 (pg/mL) | Plasma IFNα (pg/mL) | Plasma IFNγ (IU/mL) | Stimulated IL-1β (pg/mL) | Stimulated IL-6 (pg/mL) | Stimulated IFNα (pg/mL) | Stimulated IFNγ (IU/mL) |
| --- | --- | --- | --- | --- | --- | --- | --- | --- | --- | --- | --- | --- |
| 1 | Mild | 34 | M | N | 0 | 0 | 0 | 0 | 1622 | 19684 | NA | 276 |
| 2 | Mild | 39 | F | N | 0 | 0.48 | 0 | 0 | 6338 | 33841 | NA | 98.3 |
| 3 | Mild | 39 | F | N | NA | NA | 0 | 0 | 5383 | 46885 | 1876 | 2435 |
| 4 | Mild | 42 | F | Y | NA | NA | 0 | 0 | 5956 | 40667 | 293 | 537 |
| 5 | Mild | 28 | F | N | NA | NA | 0 | 0 | 5956 | 29968 | 1768 | 776 |
| 6 | Mild | 39 | F | N | 0.372 | 1.26 | 6.04 | 0 | 2528 | 28391 | 161 | 13 |
| 7 | Mild | 55 | F | Y | NA | NA | 0 | 0 | 5765 | 56043 | 674 | 79 |
| 8 | Mild | 71 | F | N | 0.728 | 1.71 | 0 | 0 | 1003 | 14654 | NA | 11.4 |
| 9 | Mild | 39 | M | N | 0.152 | 1.67 | 0 | 0 | 954 | 10863 | NA | 10 |
| 10 | Mild | 31 | M | Y | NA | NA | 0 | 0 | 4456 | 25154 | 1335 | 915 |
| 11 | Mild | 30 | F | N | NA | NA | 0 | 0 | 2818 | 31785 | 76.6 | 224 |
| 12 | Moderate | 74 | M | Y | 0.14 | 4.21 | 0 | 0 | 4116 | 71028 | 35.6 | 62.4 |
| 13 | Moderate | 56 | M | Y | 0.224 | 66.1 | 0 | 0 | 10338 | 78594 | 11.7 | 82.3 |
| 14 | Severe | 77 | M | Y | 0.734 | 85.1 | 0 | 0 | 520 | 22915 | 56.5 | 5.9 |
| 15 | Severe | 65 | M | Y | 1.1 | 34.8 | 0 | 0 | 14024 | 89843 | 0 | 0.2 |
| 16 | Severe | 53 | M | Y | 0.33 | 9.27 | 0 | 0 | 3876 | 66870 | NA | 1.8 |
| 17 | Severe | 75 | M | Y | 0.289 | 267 | 0 | 0 | 7790 | 149797 | 1.75 | 3 |
| 18 | Severe | 77 | M | Y | 0.173 | 56.1 | 0 | 0 | 1575 | 37591 | 74.3 | 12.4 |


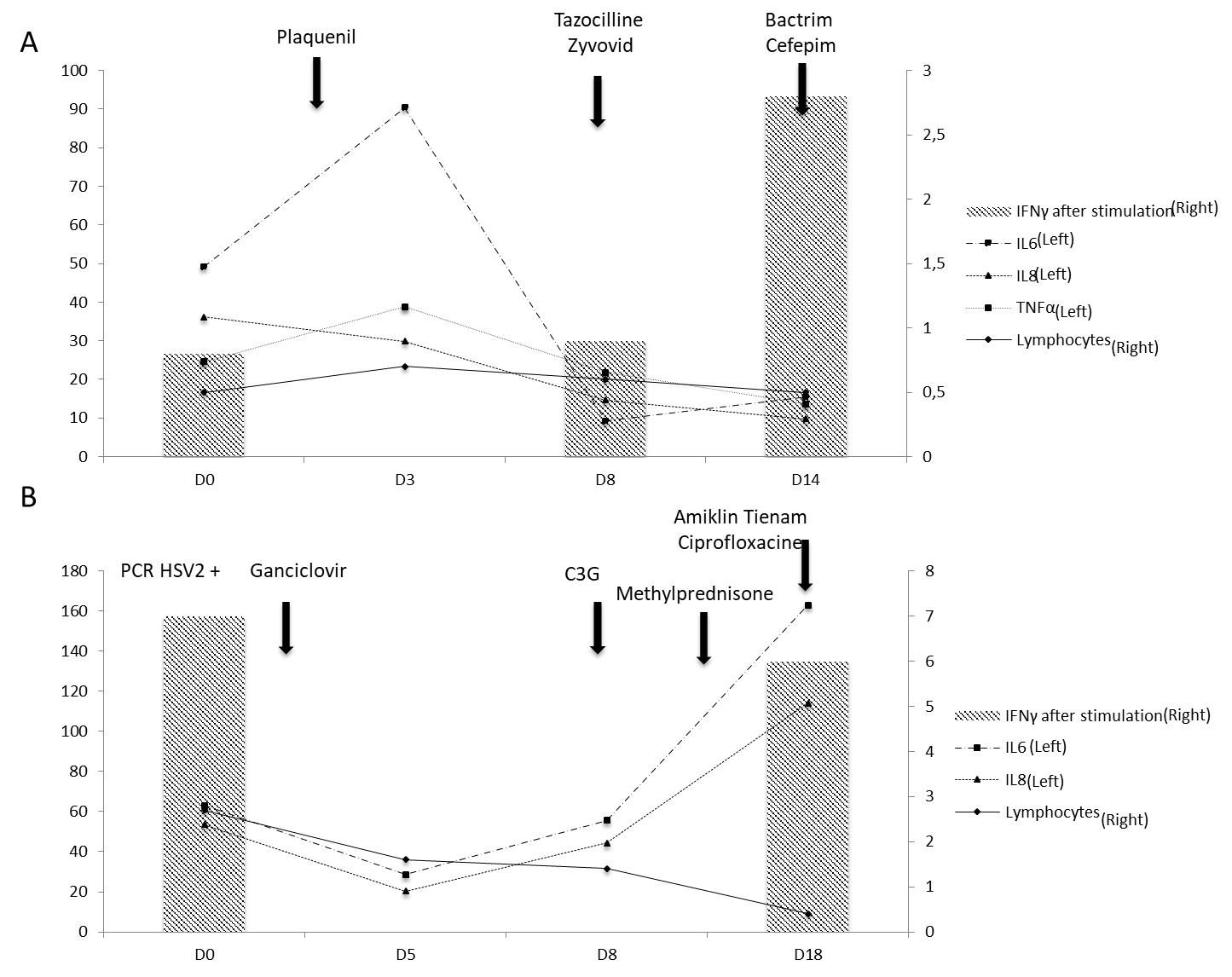


**Supplementary Figure 1: Individual evolution of non-stimulated and stimulated cytokine levels in two COVID-19 patients according to their clinical evolution.** A) Patient 1 had low stimulated IFNγ at admission to the hospital and relatively high plasma levels of inflammatory cytokines IL6, IL8 and TNFα. Upon treatment with Plaquenil and later with antibiotics the levels of plasma pro-inflammatory cytokines dropped while the level of stimulated IFNγ increased. The patient fully recovered and was discharged from the hospital. B) Patient 2 presented with similar non-stimulated plasma cytokine levels as patient 1 and similarly low levels of stimulated IFNγ. However, despite antiviral, antibiotic and corticoid treatment received his non-stimulated plasma pro-inflammatory cytokines spiraled leading to the onset of the cytokine storm while his stimulated IFNγ levels remained low, finally leading to death. C3G, 3^rd^ generation of cephalosporin; HSV2, herpes simplex virus 2; IFN, interferon; IL, interleukin; TNF, tumor necrosis factor; PCR, polymerase chain reaction.
